# A Stroll Through Saffron Fields, Cannabis Leaves, and Cherry Reveals the Path to Waste-Derived Antimicrobial Bioproducts

**DOI:** 10.3390/ph18071003

**Published:** 2025-07-03

**Authors:** Stefania Lamponi, Roberta Barletta, Michela Geminiani, Alfonso Trezza, Luisa Frusciante, Behnaz Shabab, Collins Nyaberi Nyong’a, Annalisa Santucci

**Affiliations:** 1 Department of Biotechnology, Chemistry & Pharmacy, University of Siena, Via Aldo Moro, 53100 Siena, Italy; stefania.lamponi@unisi.it (S.L.); r.barletta@student.unisi.it (R.B.); geminiani2@unisi.it (M.G.); luisa.frusciante@unisi.it (L.F.); b.shabab@student.unisi.it (B.S.); c.nyonga@student.unisi.it (C.N.N.); annalisa.santucci@unisi.it (A.S.); 2 SienabioACTIVE, University of Siena, Via Aldo Moro, 53100 Siena, Italy; 3 ARTES 4.0, Viale Rinaldo Piaggio 34, 56025 Pontedera, Italy

**Keywords:** *Cannabis sativa* L., *Crocus sativus*, *Prunus avium* L., *Staphylococcus aureus*, *Bacillus subtilis*, multiple sequence alignment, molecular modelling, MIC, molecular docking

## Abstract

**Background:** The accumulation of agri-food waste is a major environmental and economic challenge and converting these by-products into bioactive compounds fits within the circular bioeconomy. This study aimed to evaluate the antimicrobial potential of extracts derived from *Cannabis sativa* L. leaves (CSE), *Crocus sativus* tepals (CST), and *Prunus avium* L. cherry waste (VCE) against four key bacterial species (*Staphylococcus aureus*, *Bacillus subtilis*, *Escherichia coli*, and *Pseudomonas aeruginosa*). **Methods:** Minimum inhibitory concentration (MIC) assays were performed to assess antibacterial activity, while a bioinformatic pipeline was implemented to explore possible molecular targets. Full-proteome multiple sequence alignments across the bacterial strains were used to identify conserved, strain-specific proteins, and molecular docking simulations were applied to predict binding interactions between the most abundant compounds in the extracts and their targets. **Results:** CSE and CST demonstrated bacteriostatic activity against *S. aureus* and *B. subtilis* (MIC = 15.6 mg/mL), while VCE showed selective activity against *B. subtilis* (MIC = 31.5 mg/mL). CodY was identified as a putative molecular target for CSE and CST, and ChaA for VCE. Docking results supported the possibility of spontaneous binding between abundant extract constituents and the predicted targets, with high binding affinities triggering a strong interaction network with target sensing residues. **Conclusions:** This study demonstrates the antimicrobial activity of these agri-food wastes and introduces a comprehensive in vitro and in silico workflow to support the bioactivity of these agri-food wastes and repurpose them for innovative, eco-sustainable applications in the biotechnology field and beyond.

## 1. Introduction

Over the past fifty years, the global population has spiked from 2.5 to 7.9 billion, largely fueled by a linear economic paradigm centred on the extraction, transformation, consumption, and disposal of natural resources [1]. This unsustainable trajectory has led to profound environmental, economic and social repercussions. In contrast, the circular economy prioritises waste reduction, resource efficiency, and material reuse [2,3].

A particularly interesting application field for circular bioeconomy is scientific research, yielding a circular bioeconomy where sustainable products are generated from biological wastes, mostly from agri-food, industrial, and agricultural origin [4].

Food wastes have long been seen only as feed additives for animals [5], but recent and novel applications include biorefineries to produce natural renewable gas and the textile sector relying on natural dyes for tissue staining, nutraceuticals, and pharmaceuticals sectors [6,7].

Our previous studies, conducted in a circular bioeconomy setting, focused on exploring the bioactive potential of *C. sativa* L., *C. sativus*, and *P. avium* L. by-products, highlighting their antioxidant and anti-inflammatory properties [8,9,10].

*C. sativa* L., commonly known as hemp and part of the *Cannabaceae* family, is traditionally cultivated for fibre, seeds, and medicinal purposes. Despite the historical classification into multiple species, a single species is identified by modern taxonomy with distinct subspecies and chemotypes based on cannabinoid profiles: THC-dominant (Type I), CBD-dominant (Type III or industrial hemp), balanced (Type II), and rarer types such as CBG-rich (IV) or cannabinoid-free (V) [11,12]. Industrial hemp, defined by its low THC content (<1%), is unsuitable for recreational use but has been gathering attention for its role in sustainable agriculture. Its cultivation helped reduce dependence on non-renewable resources and valorised biomass-rich processing by-products such as hurds, leaves, and inflorescences [13,14]. The richness of such industrial residues in phytocannabinoids and flavonoids would indeed justify their powerful anti-inflammatory and antioxidant properties [15,16,17].

*C. sativus*, the source of saffron, belongs to the *Iridaceae* family and is the most expensive cultivated herb in the world, appreciated for its culinary and colouring properties [18]. Beyond its traditional uses in pain relief and as an herbal medicine, recent investigations highlighted the pharmacological significance of key molecules, including crocin, safranal, and kaempferol, which exhibit various bioactive properties [19,20,21]. Processing discards large volumes of floral by-products, particularly tepals, comprising undifferentiated petals and sepals and representing 80% of the floral mass [22]. Tepals are rich in bioactive antioxidant compounds, making them eligible for anti-inflammatory purposes [23,24,25].

*P. avium* L., or sweet cherry, is a widely cultivated species in the *Rosaceae* family, appreciated for its flavour and nutritional properties [26]. Increasing scientific interest has centred on its rich profile of bioactive compounds in the fruit and frequently discarded parts such as skins, seeds, stems, leaves, and flowers with antimicrobial and anti-inflammatory activities [27], drawing interest for pharmaceutical and cosmetic applications [28,29,30].

Supported by this previous research on the by-products of *C. sativa* L., *C. sativus*, and *P. avium* L. as promising sources of bioactive compounds, the present study aimed to evaluate the antimicrobial activity of our three hydroalcoholic extracts from *C. sativa* L. leaves, *C. sativus* tepals (both sourced from Tuscany, Italy), and *P. avium* L. non-compliant cherry fruits cultivated in the Italian region of Vignola and under the “Vignola Protected Geographical Indication (PGI)”, against four bacterial strains (*S. aureus*, *P. aeruginosa*, *E. coli*, *B. subtilis*) chosen as Certified Microorganisms Standards (Certified Microbiological Reference Materials) according to ISO/IEC 17025 [31].

*Cannabis sativa* leaves (CSE) and *Crocus sativus* tepals (CST) were characterised in our previous works through ultra-performance liquid chromatography coupled with tandem mass spectrometry (UPLC-MS/MS) [8,9], and *Prunus avium* cherry waste (VCE) was characterised through ultra-high performance liquid chromatography (UHPLC) combined with electrospray ionisation tandem mass spectrometry (ESI-MS/MS) [10]. A total of 88, 87, and 87 metabolites were identified in CSE, CST, and VCE, respectively, and the most abundant ones are reported in Table S1 [8,9,10].

UPLC-MS/MS CSE profiling revealed a diverse and rich composition of secondary metabolites. Among these, cannabinoids were the dominant molecular class, underscoring the characteristic profile of *C. sativa* L. extracts, whilst flavonoids were the second most abundant class [8]. The cannabinoid composition of our extract was notably distinct since the primary compound, cannabinoid acid (CBDA, c6), constituted 78.92% of the total composition, substantially higher than the 40–50% range reported in similar studies. Our extract’s tetrahydrocannabinoid acid (THCA, c3) content was 11.91%, much higher than the typical <5% found in other studies. In contrast, cannabidiol (CBD, c1) (the decarboxylated form of CBDA) represented only 3.56%, which is lower than the 10–15% levels often reported in other *C. sativa* extracts. This discrepancy suggests that our extraction process involved minimal decarboxylation, thereby maintaining a higher proportion of acidic cannabinoids like CBDA and THCA [8]. Flavonoids were also detected. Cannflavin (c5), luteolin (c8), vitexin A (c4), genistein (c7), and lucidone B (c2) were present in amounts of 1.45%, 1.09%, 0.63%, 0.57%, and 0.20%, respectively [8]. This unique and diversified chemical profile, with a clear emphasis on acidic cannabinoids like CBDA and THCA, as well as a broad spectrum of flavonoids, would strengthen the hypothesis that the cultivar selection, cultivation conditions, and extraction methods significantly influence the chemical composition of the extract [32]. This would also impact the therapeutic profile as the preservation of acidic cannabinoids and the enrichment in flavonoids may offer enhanced anti-inflammatory, antioxidant, and neuroprotective benefits [33,34,35].

From UPLC-MS/MS, it was possible to detect that CST was mostly made of flavonoids, including flavones, flavonols, and flavanones [9]. Among these, the kaempferol glycoside 3-O-sophoroside (KOS-3, s1) was identified as the most abundant and key component, which is well-documented for its antioxidant and anti-inflammatory properties [22,36,37,38]. The second most prominent compound was astragalin (kaempferol 3-O-glucoside) [9], present in a variety of medicinal plants and exhibiting broad pharmacological activities, including anti-inflammatory, antioxidant, neuroprotective, cardioprotective, anti-osteoporotic, and antitumoral effects [39]. Other notable compounds were 6-hydroxyluteolin, a hydroxy-derivative of luteolin, quercetin, and its derivative isorhamnetin [9], all extensively studied for their anti-inflammatory properties [40].

UHPLC-ESI-MS/MS analysis of VCE revealed a complex and diverse phytochemical profile, predominantly composed of flavonoids (69%), followed by fatty acids and their conjugates (13%), and phenylpropanoids [10]. This high flavonoid content aligns with prior research on the rich phenolic composition of cherry fruits and by-products, supporting these compounds’ antioxidant and anti-inflammatory properties [41,42]. The three most abundantly identified flavonoids included sakuranin (v1), aequinetin (v2), and dihydrowogonin (v3), respectively, whilst others included quercetin, catechin, and epicatechin [10]. Alongside this, subclass representatives like kaempferol 3-O-rutinoside (flavonol), catechin (flavanol), chrysin-7-O-glucoside (flavone), and sakuranetin-5-O-glucoside (flavanone) [10]. All these compounds, together with non-flavonoid phenolics, contribute to the extract’s broad bioactivity, particularly its antioxidant and anti-inflammatory effects [43,44], hinting at VCE utility for managing oxidative stress and inflammation [10]. Additionally, the literature body confirmed the antimicrobial activity of these molecules against a range of pathogens [45,46,47,48,49].

Based on such evidence, in this work, MIC assays were carried out to evaluate the potential extracts’ antimicrobial efficacy. The results revealed a bacteriostatic effect (MBC/MIC ratio > 4) of CSE and CST extracts against the Gram-positive pathogen *S. aureus* and *B. subtilis*, with a MIC value of 15.6 mg/mL. The VCE showed a selective bacteriostatic activity (MBC/MIC ratio > 4) against *B. subtilis*, with a MIC value of 31.5 mg/mL. None of the extracts impacted on the growth of *E. coli* or *P. aeruginosa*.

Such activity against *S. aureus* and/or *B. subtilis* suggests potential health-related applications for these plant-based extracts. *S. aureus* is a clinically important pathogen implicated in a broad spectrum of infections, including skin and soft tissue infections, pneumonia, and sepsis, with rising concern due to multidrug-resistant strains like MRSA [50,51]. Conversely, *B. subtilis*, though typically non-pathogenic, is frequently associated with food spoilage and is a model organism for studying microbial contamination and hygiene in food processing environments [52,53,54].

Building on the in vitro findings, an in silico pipeline was set up [55,56] to explore the extracts’ potential molecular targets and unravel their specific bioactivity. Given that CSE and CST showed comparable activity profiles against the same bacterial strains, they were grouped for in silico investigation through multiple sequence alignment analyses, and two bacterial targets of interest were pinpointed. The first, CodY, a global transcriptional regulator conserved in both *S. aureus* and *B. subtilis* but absent in all the other tested strains, accounted for the shared activity of CSE and CST. The second, ChaA, a Ca^2+^/H^+^ antiporter unique to *B. subtilis*, mirrored the selective effect of VCE. Molecular docking simulations were then performed on the most abundant compounds (c1–c8; s1; and v1–v3) in each extract to assess their interactions with the selected targets (Tables S2–S4). The docking results supported the proposed target–compound interactions elucidating the in vitro activity.

This integrated circular bioeconomy approach, combining experimental assays with a bioinformatics pipeline, suggests a potential strategy for next-generation bioproducts toward sustainable, green, and eco-friendly uses.

## 2. Results

### 2.1. In Vitro Results

#### Extract Susceptibility in Bacterial Strains

Results from the MIC assay are summarised in Table 1. Both CSE and CST extracts exhibited MIC values of 15.6 mg/mL against *S. aureus* and *B. subtilis*, suggesting a detectable antimicrobial activity against these bacterial strains.

In comparison, the VCE extract showed a higher MIC of 31.5 mg/mL against *B. subtilis*, implying that its activity is lower than that of CSE and CST. No inhibitory activity was observed for VCE against *S. aureus*, as the MIC value exceeded the highest concentration tested (>31.5 mg/mL).

None of the extract exhibited an antimicrobial activity against *E. coli* or *P. aeruginosa* as in both cases the MIC values exceeded the maximum concentration tested (>31.5 mg/mL).

To evaluate the relative potency of the natural extracts, the MIC values of tetracycline and gentamicin, used as reference antibiotics given their known efficacy [57,58,59,60], were also provided as a benchmark of comparison (Table 1).

### 2.2. In Silico Results

#### 2.2.1. Multiple Sequence Alignment and Target Detection

In vitro analysis demonstrated that *S. aureus* and *B. subtilis* were susceptible to the treatment with CSE and CST extracts, while *P. aeruginosa* and *E. coli* showed no sensitivity. Additionally, only *B. subtilis* exhibited susceptibility to VCE extract.

To identify potential molecular targets responsible for this differential susceptibility, MSAs were conducted between the protein sets of *S. aureus* and *B. subtilis* versus those of *P. aeruginosa* and *E. coli*. Homologous sequences with ≥30% identity and sufficient coverage were filtered out using a custom Python 3.0 script.

Among the remaining non-homologous sequences, a single conserved protein was identified between *S. aureus* and *B. subtilis*: the global transcriptional regulator CodY. This suggests CodY as a potential shared target for the active compounds in CSE and CST extracts.

To identify the specific target in *B. subtilis* responsible for its sensitivity to VCE, a second MSA was performed between *B. subtilis* and the other three species. Proteins with <20% identity and low coverage were retained. After removing synthetic constructs and uncharacterised proteins, the Ca^2+^/H^+^ antiporter ChaA was identified as a unique *B. subtilis* protein with no homologues in the other species, suggesting it as the probable VCE target.

#### 2.2.2. Molecular Modelling and Docking Simulations

AlphaFold generated the target 3D structures with a very high confidence, and ramachandran and chi plot analyses checked the accuracy of the predict models, confirming the accuracy of the models. Finally, MDs optimised the target structures and allowed us to obtain the most favourable conformation of the target, representing the starting conformation of the docking simulation.

CSE and CST extracts were tested against the targets of *S. aureus* and *B. subtilis* through in silico virtual screening, while VCE extract was tested against the *B. subtilis* ChaA.

#### 2.2.3. CSE Extract Against *S. aureus* and *B. subtilis* CodY

**Figure 1:**
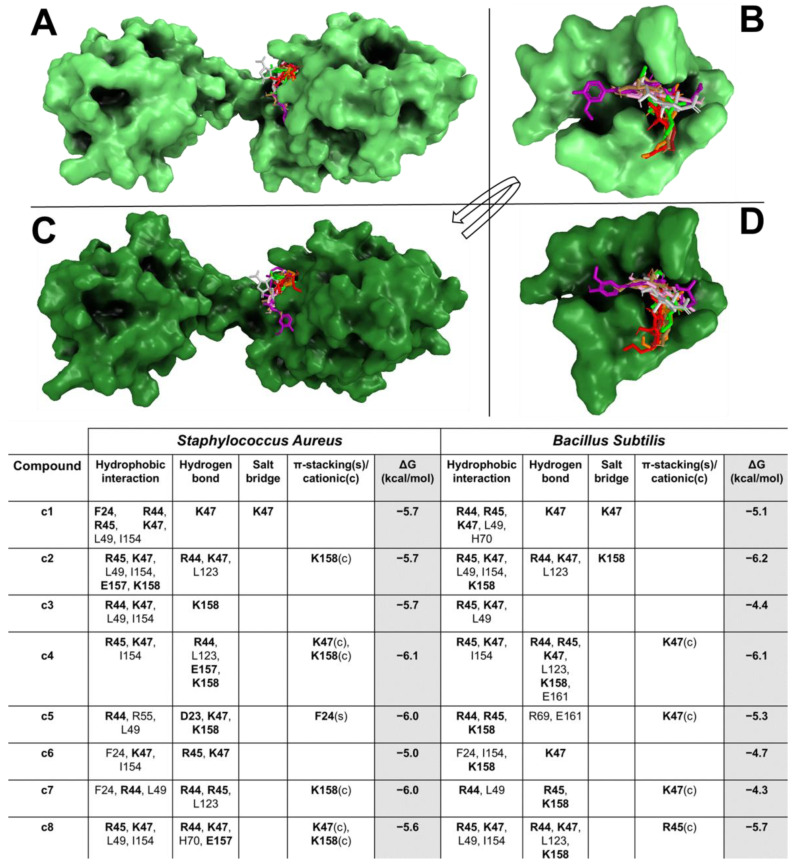
*S. aureus* and *B. subtilis* CodY/CSE and CST extract compound interaction overview. The *S. aureus* and *B. subtilis* CodY and the enlargement of the binding pockets in complexes with compounds are shown with light green (**A**,**B**) and deep green (**C**,**D**) surfaces, respectively. The compounds are reported in coloured sticks in this order: c1 (red), c2 (green), c3 (orange), c4 (golden), c5 (purple), c6 (grey), c7 (pink), and c8 (brown). The table reports all interaction networks of the compound in complex with the target. The residues marked in bold represent the key residues of the target. A white box, where no residue is reported, means that no interaction is present between residues and ligand.

The docking results showed that all compounds were able to spontaneously bind (with binding affinity from −4.3 kcal/mol to −6.2 kcal/mol) within their target binding pocket, forming a large hydrophobic interaction and hydrogen bond network (Figure 1A–D). Docking results showed that c1–c8 and s1 formed hydrogen bonds and π-stacking and/or π-stacking cationic with target critical residues, such as D23, F24, R44, R55 K47, E157, and K158.

#### 2.2.4. CST Extract Against *S. aureus* and *B. subtilis* CodY

**Figure 2:**
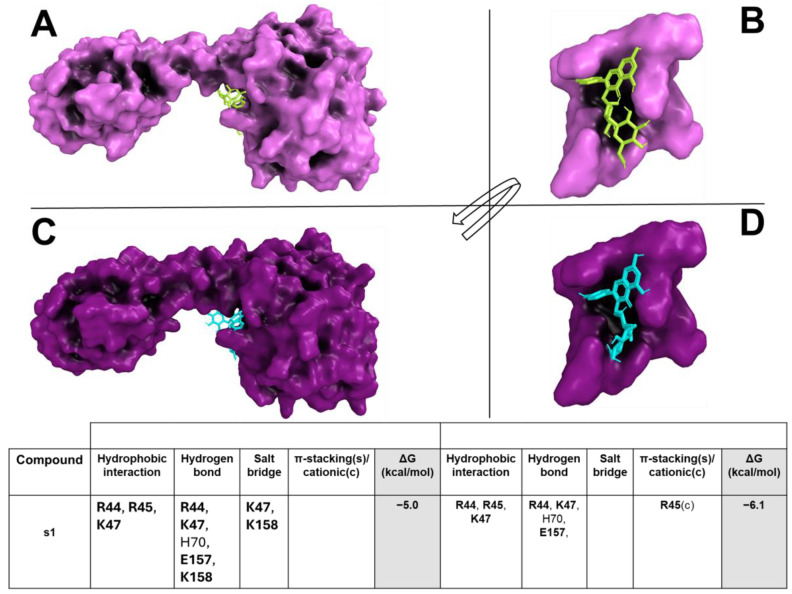
*S. aureus* and *B. subtilis* CodY/s1 (CVE extract) interaction overview. The *S. aureus* and *B. subtilis* CodY and the enlargement of the binding pockets in complex with compounds are shown with pink (**A**,**B**) and purple (**C**,**D**) surfaces, respectively. S1 is represented as a yellow (for *S. aureus*) or cyan (for *B. subtilis*) stick. The table reports all interaction networks of the compound in complex with the target. The residues marked in bold represent the key binding residues of the target. White boxes, where no residue is reported, mean that no interaction is present between residues and ligand.

The docking results showed that s1 was able to bind (with binding affinity from −4.3 kcal/mol to −6.2 kcal/mol) within the target binding pocket, forming a large hydrophobic interaction and hydrogen bond network (Figure 2A–D). s1 formed hydrogen bonds and a salt bridge with target critical residues, such as R44, R45, K47, E157, and K158.

#### 2.2.5. VCE Extracts Against *B. subtilis* ChaA

**Figure 3:**
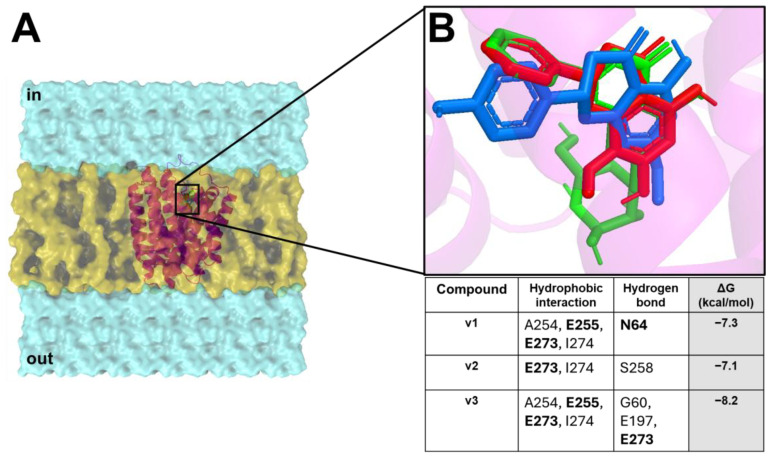
*B. subtilis* ChaA/VCE extract compound interaction overview. (**A**) *B. subtilis* ChaA 3D structure (magenta cartoon) inside the bilayer (yellow surface), and the cytoplasm and extracellular side (cyan surface). (**B**) Enlargement of the binding pocket in complex with v1 (red stick), v2 (blue stick), and v3 (green stick). The table reports all interaction networks of the compound in complex with the target. The residues marked in bold represent the key binding residues of the target.

The docking results showed that all compounds were able to spontaneously bind (with binding affinity from −7.1 kcal/mol to −8.2 kcal/mol) within the target binding pocket, exhibiting a wide hydrophobic and polar interaction network (Figure 3).

In silico docking showed that v1–v3 formed hydrophobic interactions and hydrogen bonds with the target key residues N64, E255, and E273.

## 3. Discussion

The circular economy is a sustainable alternative to the traditional linear model, which relies on extraction, use, and resource disposal [3]. It focuses on extending resource life through reuse, recycling, and regeneration, addressing concerns like resource depletion and environmental degradation [61]. This model is key to tackling global environmental challenges by reducing waste and promoting sustainable practices, rendering it eligible for industries looking to reduce carbon footprints and support economic growth [62].

The relevance of the circular economy mostly stands out in sectors like agriculture, where agri-food wastes can become valuable resources. Closing the loop in food production and processing indeed fosters innovation and encourages responsible resource use. This approach is the circular bioeconomy, which utilises biological resources to produce bio-based products and renewable energy [63]. This model is particularly compelling when considering the vast amount of biological waste generated by industries. The valorisation of agri-food waste, such as crop residues, food by-products, and plant biomass, emerged as a central strategy in this field. Through bioconversion, fermentation, and enzymatic hydrolysis, agricultural waste can be transformed into high-value products such as bioplastics, biofuels, and natural ingredients for pharmaceuticals and cosmetics [64].

This approach presents several benefits: waste minimisation, reduced dependence on fossil-based resources, and creation of new opportunities for economic growth. Furthermore, by exploiting biological waste, industries can tackle major global challenges such as food security, climate change, and the depletion of non-renewable resources [65].

Plants have long been recognised as a good source of bioactive compounds with diverse biological activities suitable for health-related applications. These compounds, such as polyphenols, flavonoids, alkaloids, and terpenoids, were studied for their antioxidant, anti-inflammatory, and antimicrobial properties [34]. Outstanding examples of medicinal plants include turmeric, whose bioactive compound curcumin is well-known for its potent antioxidant and anti-inflammatory properties [66]; garlic, where the strong antimicrobial effects are attributed to the compound allicin [67]; and green tea, which is rich in polyphenols, offering significant antioxidant benefits [68].

In the context of the circular bioeconomy, the focus shifted to plant-based extracts derived mostly from agri-food waste, which hold great promise as a sustainable source of bioactive compounds. For instance, grape pomace, a by-product of winemaking, contains polyphenols with antioxidant and antimicrobial properties [69] and olive leaves, often discarded in olive oil production, yield oleuropein, an antimicrobial and anti-inflammatory molecule [70].

The corresponding extracts derived from *C. sativa* L. leaves, *C. sativus* tepals, and *P. avium* cherry waste obtained and characterised in our previous works, highlighted distinct and rich compositional profiles reflective of their botanical origins and extraction parameters [8,9,10]. To generate CSE, CST, and VCE, the respective biomasses were collected, ground, and an extraction with an ethanol–water (70:30 *v*/*v*) mixture was carried out. In compliance with EU regulations (Directive 2009/32/EC), ethanol is recognised as a safe solvent for food and nutraceutical applications. Then, the supernatant was separated from the residual biomass, filtered, and subjected to rotary evaporation to remove the organic solvent. Finally, the aqueous residue was freeze-dried [8,9,10].

Characterisation showed how CSE was notably rich in secondary metabolites, primarily cannabinoids, with CBDA as the predominant compound, followed by THCA and minor levels of CBD, suggesting minimal decarboxylation and emphasising the extract’s preservation of acidic cannabinoids. Flavonoids (cannflavin A, luteolin, vitexin, genistein, and lucidone B) were the second most abundant molecular class [8]. Cannabinoids are well documented for their anti-inflammatory properties [15,16].

CST was instead mostly made of flavonoids, such as flavones, flavonols, and flavanones. KOS-3 was the principal component, but the extract also contained significant levels of astragalin, 6-hydroxyluteolin, genistein, and quercetin derivatives, indicating a broad flavonoid spectrum typical of saffron floral waste [9]. Flavonoids were long prized for their wide range of properties, including antioxidant and antimicrobial ones [23,24,25,71,72].

Lastly, VCE showed a 60% flavonoid content, featuring sakuranin, aequinetin, and dihydrowogonin as major constituents [10]. However, fatty acids and their conjugates and phenylpropanoids (C6–C3) were also detected [10]. All these compounds are responsible for the observed antioxidant and anti-inflammatory activities of the extract [43,44].

Building on the previously demonstrated antimicrobial activity of these extracts against various pathogens [27,73,74], this study aimed to enlarge the literature body on their antibacterial properties and possible applications. Hence, the activity of CSE, CST, and VCE was evaluated against four bacteria (*S. aureus*, *P. aeruginosa*, *E. coli*, and *B. subtilis*) through a comprehensive in vitro and in silico approach.

Results from the MIC assay indicated that both CSE and CST exhibited bacteriostatic activity against *S. aureus* and *B. subtilis*, with MIC values of 15.6 mg/mL. In contrast, VCE demonstrated a significantly higher MIC of 31.5 mg/mL against *B. subtilis*, suggesting a lower activity than CSE and CST. Furthermore, VCE did not exhibit any efficacy against *S. aureus*, as its MIC value exceeded the highest concentration tested (>31.5 mg/mL), indicating a lack of potency against this strain. These findings highlighted the different proprieties of the extracts, with CSE and CST displaying superior activity against *S. aureus* and *B. subtilis* compared to VCE. The comparison with tetracycline and gentamicin as reference antibiotics provided a valuable context for evaluating the relative antimicrobial potency of these natural extracts. The MIC values for tetracycline and gentamicin were consistent with its known effectiveness against these pathogens, offering a benchmark for comparison [57,58,59,60].

Based on in vitro results, a bioinformatics pipeline was developed to dissect the extract activity against the bacterial strains used in this study. In detail, CSE and CST exhibited bacteriostatic activity against *S. aureus* and *B. subtilis*, differently from VCE which revealed activity only for *B. subtilis*. Given such evidence, to identify the potential extracts compound/s against the bacteria target/s, an MSA was performed. The criteria were based on the differing activity of the extracts against the bacteria strains. In depth, for CSE and CST extracts, our approach was to identify all homologue biological targets shared between *S. aureus* and *B. subtilis* (where the extracts are active), excluding all homologue biological targets shared with *E. coli* and *P. aeruginosa* (where the extracts are not active). Firstly, the MSA was performed among the protein sequences available in NCBI Datasets of *S. aureus* against *E. coli* and *P. aeruginosa* (excluding all the homologue protein sequences available in NCBI Datasets of *B. subtilis* against *E. coli* and *P. aeruginosa*, excluding all the homologue proteins). Thus, considering the remaining proteins of *S. aureus* and *B. subtilis*, another MSA was carried out and all non-homologue proteins were discarded, obtaining only the homologue proteins shared between *S. aureus* and *B. subtilis*. Finally, excluding from the list all synthetic constructs and hypothetical and putative proteins, we obtained one only target which satisfied our criteria for *S. aureus* and *B. subtilis*: the CodY protein, a pleiotropic transcription factor CodY with a key role in integrating metabolism and virulence factor expression. A similar approach was used for the VCE extract; thus, the MSA was performed among the protein sequences available in NCBI Datasets of *B. subtilis* against *S. aureus*, *E. coli*, and *P. aeruginosa* (excluding all the homologue proteins, synthetic constructs, hypothetical, and putative proteins), obtaining one only target, named ChaA. To identify the compounds of the extracts potentially active against the CodY (CSE and CST extracts) and ChaA (VCE extract), a docking simulation was performed between the targets and extract compounds. To reinforce and confer robustness and reliability to our simulations, the docking was carried in a critical binding region of the targets. In CodY, the docking simulation was focused on a binding region able to bind a wide range of substrates, which, upon ligand binding, is involved in conformational changes that propagate to the homodimer interface and reorient the linker helices and DNA binding domains, leading to transcriptional alterations [75]. Docking results showed the ability of the compounds to strongly bind in this binding region, forming a wide hydrophobic and polar interactions with the target binding residues located in this region and involved in the conformational change in CodY with a potential modification of the binding on the DNA interface, modifying transcriptional mechanisms [75]. C1, c2, c4, c5, c7, c8, and s1 formed hydrogen bonds and π-stacking and/or π-stacking cationic with target critical residues, such as D23, F24, R44, R55 K47, E157, and K158 [75], suggesting their potential ability to bind and influence the biological function of the target. Similarly, VCE extract compounds bound in a sensing binding region of *B. subtilis* ChaA, H^+^/Ca^2+^ (calcium ion) antiporter (CAX), which plays an important role in maintaining cellular Ca2+ homeostasis in bacteria by promoting Ca^2+^ efflux across the cell membranes [76]. The compounds bound with a very high binding affinity trigger a large hydrophobic and polar interaction network with key residues in a critical region of the target (mini-sensor region), involved in the Ca^2+^ binding and the in/activation of the channel [76]. The binding of our compounds and their interactions would suggest a potential disruptor effect in this region with alterations about its biological function, explaining their in vitro activity.

This study highlighted the potential of plant-based extracts derived from agricultural waste as sources of antimicrobial compounds with a modest but still detectable antimicrobial activity.

Molecular docking reinforced in vitro findings and was employed as a preliminary, hypothesis-generating approach to explore potential interactions between major metabolites in the extracts and conserved bacterial protein targets identified through a comparative bioinformatics pipeline. Importantly, the docking data presented here are not intended as conclusive validation of molecular mechanisms but rather to contextualise and support the observed in vitro antimicrobial activity.

The primary aim of this work remains not to isolate or characterise individual antimicrobial agents, but rather to evaluate the biological potential of whole plant-based extracts in a circular bioeconomy framework. Nevertheless, this integrative approach combining in vitro assays and in silico analysis offers a valuable foundation for future research into bio-waste-derived antimicrobial applications.

Importantly, the aim of this study was not to propose new antibiotics, as the MIC values observed in this study, ranging from 15.6 mg/mL to >31.5 mg/mL, are significantly higher than those typically reported for conventional antibiotics, which often act in the μg/mL range. This contrast is expected and well-documented in natural product research, where crude or semi-purified materials frequently show activity only at higher concentrations [77,78]. Importantly, this work aimed to evaluate the potential reutilisation of agricultural by-products within a circular bioeconomy framework. From this perspective, even modest antimicrobial activity is interesting, as it supports the concept of valorising waste materials for functional applications, such as hygiene products, surface treatments, or preservatives. This would contribute to environmental sustainability and reduce resource loss and pollution [3,61].

## 4. Materials and Methods

### 4.1. Materials

*S. aureus* WDCM 00032, *P. aeruginosa* WDCM 00025, *E. coli* WDCM 00090, and *B. subtilis* WDCM 00003 Vitroids™ (Sigma-Aldrich, Saint Louis, MO, USA) Certified Microorganism Standards (Certified Microbiological Reference Materials), as well as all the materials used for bacterial tests, were purchased from Merck Life Science S.r.l. (Milan, Italy).

### 4.2. In Vitro Methods

#### 4.2.1. Preparation of CSE, CST, and VCE

The extracts were obtained and characterised as reported in our previous works [8,9,10].

In late April 2021, *Cannabis sativa* L. (Futura 75 cultivar) was cultivated on loamy soil near Siena, Italy, and harvested by the end of August. Leaves were manually separated from stems, stored in vacuum-sealed bags at 4 °C, and transported for laboratory processing. After removing visible impurities, the leaves were dried at 40 °C until a stable weight was reached, then finely ground and sieved (250 μm). Extraction was conducted by heating the powdered leaves in a 70:30 ethanol–water solution (1:10 *w*/*v*) for three hours at 80 °C. The liquid phase was filtered, concentrated via rotary evaporation, and freeze-dried, resulting in a dry extract yield of approximately 13.1% (*w*/*w*). A 100 mg/mL stock solution of CSE was prepared in DMSO and stored at −32 °C for further use [8].

Tepals from *Crocus sativus* were collected from La Scoscesa farm, located in Tuscany’s Chianti region. After harvesting, the floral parts were carefully washed and air-dried at room temperature until their weight stabilised. The dried tepals were then finely ground and subjected to extraction using a 70:30 ethanol–water solution (*v*/*v*) at 80 °C for three hours, maintaining a 1:10 (g/mL) sample-to-solvent ratio. The mixture was then centrifuged and filtered to remove solid residues. The ethanol component was evaporated under reduced pressure, and the aqueous phase was freeze-dried to obtain the dry extract. This procedure was performed in duplicate. For storage and later use, 100 mg of the extract was dissolved in 1 mL of pure DMSO to produce a 100 mg/mL CST stock solution, which was stored at −32 °C [9].

Post-processing waste from *Prunus avium* L. cherries, certified under the Vignola PGI label, was sourced after industrial sorting. This material, which included whole non-compliant fruits with pits and stems, was freeze-dried and ground into a fine powder. A 10 g portion of the powder underwent extraction via heat reflux in 100 mL of 70:30 ethanol–water (*v*/*v*) at 80 °C for three hours with constant stirring. The extract was then separated by centrifugation and filtration, and the solvent was removed through rotary evaporation. The remaining aqueous phase was freeze-dried to yield the VCE. The dry extract was stored in amber vials at −20 °C, protected from light and moisture to preserve stability [10].

All extraction procedures were carried out using a 70:30 ethanol–water solution at a 1:10 (*w*/*v*) ratio. This solvent system was selected for its proven efficiency in extracting both phenolic compounds and cannabinoids. Ethanol (Sigma-Aldrich, St. Louis, MO, USA), due to its polarity, is effective at solubilizing a broad spectrum of bioactive molecules, while the addition of water improves solvent penetration into plant tissues, thereby enhancing overall extraction yield. The 1:10 (*w*/*v*) ratio was chosen to achieve a practical yet sufficiently concentrated extract, optimising the recovery of target compounds without compromising process manageability [79,80,81].

#### 4.2.2. In Vitro Antibacterial Susceptibility Testing

The bacterial strains *S. aureus* WDCM 00032, *P. aeruginosa* WDCM 00025, *E. coli* WDCM 00090, and *B. subtilis* WDCM 00003 Vitroids™ were employed in the study using Vitroids™ containing colony-forming units (CFUs) ranging from 80 to 130 CFUs per disc. These Vitroids™ were certified according to ISO/IEC 17025 and manufactured under reproducible conditions in compliance with ISO 17034 [82], using authenticated strains obtained from the Spanish Type Culture Collection (CECT^®^). Each Vitroid™ originated from a traceable freeze-dried culture supplied by CECT^®^ (Paterna, Spain).

The inoculation procedure for each bacterial strain was conducted as follows:

1. Vitroid™ tubes were removed from the freezer and allowed to equilibrate to room temperature (5–10 min);
2. The tubes were opened, and discs were dispensed by inverting the tubes over Tryptic Soy Agar (TSA) plates (9 mm diameter);
3. Discs were left on the agar surface at ambient temperature to allow rehydration;
4. Once fully rehydrated and dissolved (approximately 10–15 min), the resulting droplets were gently spread across the plate surface, taking care to avoid over-spreading that could compromise cell viability;
5. Plates were incubated at 37 °C for 24 h. The plant extracts were initially resuspended in sterile phosphate-buffered saline (PBS) to a concentration of 300 mg/mL, then further diluted in Tryptic Soy Broth (TSB) for experimental use. Minimum inhibitory concentrations (MICs) were determined using the broth microdilution method, following Clinical Laboratory Standards Institute (CLSI, 2021) guidelines. Each assay was performed in two replicates with each concentration tested in eight replicates, with a final bacterial inoculum of 5 × 10^4^ CFUs per well. Serial two-fold dilutions of each extract were freshly prepared in 96-well microplates, using a final volume of 25 μL per well. Subsequently, 175 μL of bacterial suspension (5 × 10^6^ CFU/mL) was added to each well, yielding the desired final inoculum. Control wells containing only TSB served as growth controls, confirming the viability of the bacterial strains in the absence of inhibitory substances. MIC values were recorded after 18 h of incubation at 37 °C. Tetracycline, used as a reference antibiotic for *S. aureus* and *B. subtilis*, and gentamicin, used as a reference for *P. aeruginosa* and *E. coli*, were tested in parallel at concentrations ranging from 0.01 to 10 μg/mL.

### 4.3. In Silico Methods

#### 4.3.1. Sequence Resources and Multiple Sequence Alignment

Protein sequences of four bacterial strains were retrieved from the NCBI Datasets [83] (genome section) in FASTA format: *Escherichia coli* (Genome assembly ASM584v2; RefSeq GCF_000005845.2; 4298 protein sequences), *Staphylococcus aureus* (ASM1342v1; GCF_000013425.1; 2767 protein sequences), *Bacillus subtilis* (ASM904v1; GCF_000009045.1; 4243 protein sequences), and *Pseudomonas aeruginosa* (ASM676v1; GCF_000006765.1; 5571 protein sequences).

Multiple sequence alignment (MSA) was performed using BLAST+ v2.15.0 [84] employing the BLOSUM80 substitution matrix, a word size of 2, and an E-value threshold of ≤1 × 10^−5^. Additional parameters included -sorthits 3 and -sorthsps 0 to facilitate downstream analysis. All other settings were used at default.

An in-house Python script was developed to identify homologous proteins shared between *S. aureus* and *B. subtilis* while excluding those shared with *E. coli* and *P. aeruginosa*. Proteins with ≥30% sequence identity and sufficient coverage were considered homologous. Unique proteins in *B. subtilis* were defined as those with no detectable homology to any proteins in the other three species.

This analysis identified one homologous protein shared between *S. aureus* and *B. subtilis*: the global transcriptional regulator CodY, with UniProtKB/Swiss-Prot [85] entries Q2FZ27 (*S. aureus*) and P39779 (*B. subtilis*). Furthermore, a unique *B. subtilis* protein with no homology to proteins in the other species was identified: the Ca^2+^/H^+^ antiporter ChaA (UniProtKB/Swiss-Prot: O34840).

#### 4.3.2. Molecular Modelling and Structure Optimisation

The 3D structures of CodY from *S. aureus* and *B. subtilis*, and of ChaA from *B. subtilis*, were predicted using AlphaFold2 [86]. The predicted structures exhibited high per-residue confidence scores (pLDDT), indicating the high reliability of the models.

Structural validation was performed using PROCHECK v2.3 [87] through Ramachandran and Chi plot analyses. Energy minimization and molecular dynamics (MD) simulations were carried out using GROMACS v2019.3 [88]. CHARMM-GUI v.3.8 was used for parameter assignment and membrane bilayer construction for ChaA [89,90,91]. Each structure was solvated in a TIP3P water box and neutralised with counter-ions. Energy minimisation was performed using the steepest descent algorithm for 10,000 steps until the maximum force dropped below 100 kJ/mol/nm.

MD simulations were executed for 100 ns with a 2 fs timestep. A V-rescale thermostat [92] maintained the temperature at 300 K, and a Nose–Hoover barostat [93,94] maintained the pressure at 1 atm. The LINCS algorithm [95] constrained hydrogen-involved bond lengths. The most stable frame from the trajectory was extracted and used for docking simulations.

#### 4.3.3. Target Preparation and In Silico Docking

Ligand-target docking simulations were conducted between selected natural compounds and the identified bacterial targets. The bioactive compounds were derived from hemp (CSE), cherry (VCE), and saffron (CST) extracts. In detail, the 3D structures of cannflavin A (CID: 10071695), luteolin (CID: 5280445), vitexin (CID: 5280441), genistein (CID: 5280961), cannabidiolic acid (CID: 160570), Δ9-tetrahydrocannabinolic acid (CID: 98523), cannabidiol (CID: 644019), and lucidone B (CID: 14109411) (CSE); and Kaempferol 3-O-sophoroside (Compound CID: 5282155) (CST), and Sakuratenin (CID: 73571), aequinetin (CID: 15558425), and dihydrowogonin (CID: 11491431) (VCE) were obtained in sdf format through the PubChem database [96].

Docking was performed using AutoDock/Vina XB v.1.1.2 [97] via DockingPie 2.0 [98] implemented in PyMOL 3.0 (The PyMOL Molecular Graphics System, Version 1.2r3pre, Schrödinger, LLC, New York, NY, USA). The box with a dimension of 20 × 20 × 20 Å was defined around the putative binding site on each target [75,76]. The exhaustiveness was set to 32 and docking poses within 2 Å RMSD of the top-ranked pose were retained. All other parameters were set to default.

Protein–ligand interaction networks were generated using PLIP v1.1.0 [99,100]. Visualisation of structures and interaction interfaces was performed using PyMOL 3.0.

All simulations and analyses were conducted on a high-performance workstation equipped with an AMD Ryzen Threadripper PRO 5965WX 24-Core CPU (4.5 GHz, 140 MB cache), 128 GB DDR4 RAM (3200 MHz) (AMD, Santa Clara, CA, USA), an NVIDIA GeForce RTX 4090 GPU (24 GB) (NVIDIA, Santa Clara, CA, USA), and a storage system comprising a 2 TB NVMe SSD and dual 14 TB SATA3 HDDs, running Ubuntu 14.04.

## 5. Conclusions

This study was carried out within the circular bioeconomy to explore the potential of waste-derived by-products for potential antimicrobial applications, enhance the value of agri-food waste and expand the knowledge and uses of natural resources that are under-explored. The activity of *C. sativa* L., *C. sativus*, and *P. avium* L. extracts was assessed through a joint in vitro and in silico workflow.

In vitro MIC assays revealed that *C. sativa* L. and *C. sativus* extracts exhibited bacteriostatic activity against the relevant *B. subtilis* and *S. aureus*, with a MIC value of 15.6 mg/mL. *P. avium* L. extract was also bacteriostatic, but only on *B. subtilis*, exhibiting a MIC value of 31.5 mg/mL. Relying on such outcomes, an in silico approach was adopted to pinpoint two separated predicted bacterial targets: CodY, accounting for the activity of CSE and CST on both *B. subtilis* and *S. aureus*, and ChaA, reflecting the sole activity of VCE against *B. subtilis*. The interactions of both targets with the extracts were evaluated through molecular docking simulations, revealing strong binding affinities and wide interaction networks with critical residues of the biological targets, suggesting the potential activity of compounds against the bacterial strains. Notably, this study was conducted with the primary aim of exploring the repurposing of whole plant extracts from agri-food by-products rather than isolating or developing novel antimicrobial drugs. Given the observed antimicrobial activity against foodborne and surface-associated pathogens, the findings suggest that these extracts may hold promise for use in natural food preservation or environmental hygiene applications.

The approach and the evidence adopted here and provided in this study could be useful for future for more eco-friendly and cost-effective strategies to develop waste-derived bioproducts for different purposes.

Future studies will aim to expand antimicrobial testing to include a broader panel of foodborne and surface-associated microorganisms.

## Figures and Tables

**Table 1:** Determination of MIC values for VCE, CSE, and CST extracts against *S. aureus*, *B. subtilis*, *P. aeruginosa*, and *E. coli*. MIC values of the reference antibiotics tetracycline (which exhibits the expected activity against Gram-positive organisms) and gentamicin (active against Gram-negative ones) are also reported. The tests were performed in duplicate, with each concentration tested in eight replicates.

	MIC			
Bacterial Strain	VCE (mg/mL)	CSE (mg/mL)	CST (mg/mL)	Tetracycline/Gentamicin (μg/mL)
*S. aureus*	>31.5	15.6	15.6	4
*B. subtilis*	31.5	15.6	15.6	0.1
*P. aeruginosa*	>31.5	>31.5	>31.5	2
*E. coli*	>31.5	>31.5	>31.5	8

## Data Availability

Data is contained in the paper.

## Supplementary Materials

The following supporting information can be downloaded at: https://www.mdpi.com/article/10.3390/ph18071003/s1, Table S1: Metabolite name, PubChem CID, and 2D structure of the metabolites present in CSE, CST, and VCE and subjected to docking simulations; Table S2: Most representative matched metabolites in *C. sativa* leaves extract (CSE) and their formulas. Compounds with an area % <0.1 were considered traces; Table S3: Most representative matched metabolites in *C. sativus* tepals extract (CST) and their formulas. Compounds with an area % <0.1 were considered traces; Table S4: Most representative matched metabolites in *P.avium* extract (VCE) and their formulas. Compounds with an area % <0.1 were considered traces.
