# Supplementary material for: A Stroll Through Saffron Fields, Cannabis Leaves, and Cherry Reveals the Path to Waste-Derived Antimicrobial Bioproducts
Source: Pharmaceuticals (Basel). 2025 Jul 3;18(7):1003. doi: 10.3390/ph18071003 (PMC12299262; doi:10.3390/ph18071003)
Supplement: Supplementary file 1 [file pharmaceuticals-18-01003-s001.zip › pharmaceuticals-3682560-supplementary.pdf]

Name	PubChem CID	2D Structure
Cannflavin A (CSE)	10071695	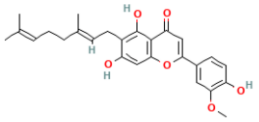
Luteolin (CSE)	5280445	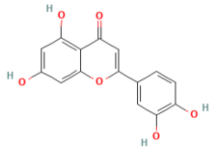
Vitexin (CSE)	5280441	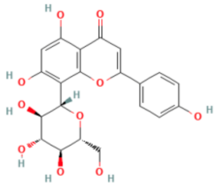
Genistein (CSE)	5280961	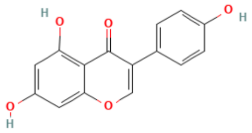
Cannabidiolic acid (CSE)	160570	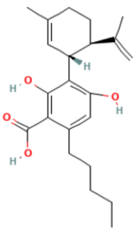
Δ9-tetrahydrocannabinolic acid (CSE)	98523	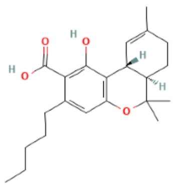

Cannabidiol (CSE)	644019	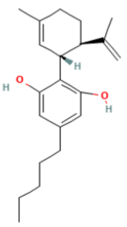
Lucidone B (CSE)	14109411	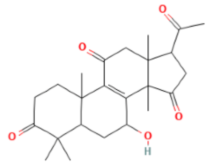
Kaempferol 3-O-sophoroside (CST)	5282155	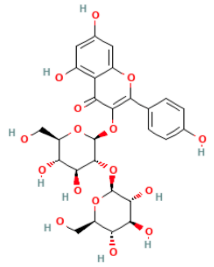
Sakuratenin (VCE)	73571	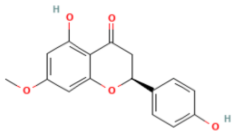
Aequinetin (VCE)	15558425	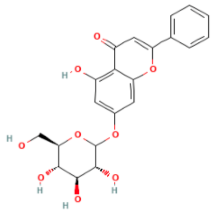
Dihydrowogonin (VCE)	11491431	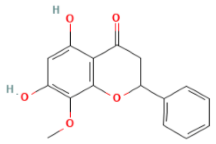

Table S1. Metabolite name, PubChem CID, and 2D structure of the metabolites present in CSE, CST, and VCE and subjected to docking simulations.

Name	Formula
Cannabidiolic acid	$C_{22}H_{30}O_4$
Δ^9-tetrahydrocannabinolic acid	$C_{22}H_{30}O_4$
Cannabidiol	$C_{21}H_{30}O_2$
Cannflavin A	$C_{26}H_{28}O_2$
Luteolin	$C_{15}H_{10}O_6$
Vitexin	$C_{21}H_{20}O_{10}$
Genistein	$C_{15}H_{10}O_5$
Lucidone B	$C_{24}H_{32}O_5$

Table S2. Most representative matched metabolites in *C. sativa* leaves extract (CSE) and their formulas. Compounds with an area % <0.1 were considered traces.

Name	Formula
Kaempferol 3-O-sophoroside	$C_{27}H_{30}O_{16}$
Astragalin	$C_{21}H_{20}O_{11}$

Kaempferol	$C_{15}H_{10}O_6$
6-Hydroxyluteolin	$C_{15}H_{10}O_7$
Isorhamnetin 3,4′-diglucoside	$C_{28}H_{32}O_{17}$
Adenosine	$C_{10}H_{13}N_5O_4$
Isorhamnetin 3-O-robinobioside	$C_{28}H_{32}O_{16}$
Quercetin-3-O-glucoside	$C_{21}H_{20}O_{12}$
Kaempferide	$C_{16}H_{12}O_6$
Apigenin 7-sophoroside	$C_{27}H_{30}O_{15}$
Daidzein	$C_{15}H_{10}O_4$
9,10-Dihydro-3,8-dihydroxy-1-methyl-9,10-dioxo-2-anthracenecarboxylic acid	$C_{16}H_{10}O_6$
Crocin 3	$C_{32}H_{44}O_{14}$
Quercetin 3-O-gentiobioside	$C_{27}H_{30}O_{17}$
Quercetin	$C_{15}H_{10}O_7$
Kaempferol 3,7,4′-triglucoside	$C_{33}H_{40}O_{21}$
Genistein	$C_{15}H_{10}O_5$
Perlolyrin	$C_{16}H_{12}N_2O_2$
Myricetin	$C_{15}H_{10}O_8$

Safranal	$C_{10}H_4O$
Apigenin	$C_{15}H_{10}O_5$
3-Hydroxy-beta-ionone	$C_{13}H_{20}O_2$
Eriodictyol	$C_{15}H_{12}O_6$

Table S3. Most representative matched metabolites in *C. sativus* tepals extract (CST) and their formulas. Compounds with an area % <0.1 were considered traces.

**Name**	**Formula**
Sakuranin	$C_{22}H_{24}O_{10}$
Aequinetin	$C_{21}H_{20}O_{09}$
Dihydrowogonin	$C_{16}H_{14}O_5$
2,3-Oxiranedioctanoic acid	$C_{18}H_{32}O_5$
Quercetin 3-rutinoside	$C_{27}H_{30}O_{16}$
Quercetin	$C_{15}H_{10}O_7$
Kaempferol 3-rutinoside	$C_{27}H_{30}O_{15}$
Pelagronidin	$C_{15}H_{11}O_5$
Chrysin	$C_{15}H_{10}O_4$
4-Ethylcatechol	$C_8H_{10}O_2$

4-O-p-Coumaroylquinic acid	$C_{16}H_{18}O_8$
9,10,18-Trihydroxyoctadecanoic acid	$C_{18}H_{36}O_5$
4-Ethylphenol	$C_8H_{10}O$
Naringenin 7-O-beta-D-glucoside	$C_{21}H_{22}O_{10}$
(-)-Epicatechin	$C_{14}H_{15}O_6$
(+)-Naringenin	$C_{15}H_{12}O_5$
Quercetin 3-rutinoside-4′-glucoside	$C_{33}H_{40}O_{21}$
Genistein 7-O-glucoside	$C_{21}H_{20}O_{10}$
Oleanolic acid	$C_{30}H_{48}O_3$
Spiraeoside	$C_{21}H_{20}O_{12}$
Linoleic acid	$C_{18}H_{32}O_2$
p-Coumaric acid 4-O-glucoside	$C_{15}H_{18}O_8$
Dihydroquercetin	$C_{15}H_{12}O_7$
16-Hydroxyhexadecanoic acid	$C_{16}H_{32}O_3$
Isorhoifolin	$C_{27}H_{30}O_{14}$
Apigenin	$C_{15}H_{10}O_5$
Dihydromyricetin 3-O-rhamnoside	$C_{21}H_{22}O_{12}$
Diosmin	$C_{28}H_{32}O_{15}$

7-Hydroxysecoisolariciresinol	$C_{22}H_{30}O_5$
3-Methylcatechol	$C_7H_8O_2$
3-O-Feruloylquinic acid	$C_{17}H_{20}O_9$
Oleic acid	$C_{18}H_{34}O_2$
Biochanin A	$C_{16}H_{12}O_5$
Malvidin 3-O-glucoside	$C_{23}H_{25}O_{12}$
Benzenemethanol	C_7H_8O
Caffeic acid 4-O-glucoside	$C_{15}H_{32}O_2$
Palmitic acid	$C_{16}H_{32}O_2$
(Z)-beta-Damascenone	$C_{13}H_{18}O$
5-Caffeoylquinic acid	$C_{16}H_{18}O_9$
Dihydrochrysin	$C_{15}H_{12}O_4$
Dicaffeoylquinic acid	$C_{25}H_{24}O_{12}$
5-Pentadecylresorcinol	$C_{21}H_{36}O_2$
p-Cymen-8-ol	$C_{10}H_{14}O$
Gibberellin A5	$C_{19}H_{22}O_5$
7-O-Methylaromadendrin	$C_{16}H_{14}O_6$
Pelargonidin 3-O-galactoside	$C_{21}H_{21}O_{10}$
Dihydroquercetin 3-O-rhamnoside	$C_{21}H_{22}O_{11}$

6-Methoxykaempferol	$C_{16}H_{12}O_7$
9,10-Epoxy-18-hydroxy-octadecanoic acid	$C_{18}H_{34}O_4$
Isorhamnetin 3-O-rutinoside	$C_{28}H_{32}O_{16}$
Ellagic acid	$C_{14}H_{16}O_8$
2,3-Dihydro-2,5,7-trihydroxy-2-(4-hydroxyphenyl)-4H-1-benzopyran-4-one	$C_{15}H_{12}O_6$
Kaempferol 4′-glucoside	$C_{21}H_{20}O_{11}$
Kaempferol 3-rutinoside-4′-glucoside	$C_{33}H_{40}O_{20}$
Cinnamic acid	$C_9H_{30}O_4$
Hexadecane-1,16-dioic acid	$C_{16}H_{30}O_4$
p-Coumaric acid ethyl ester	$C_{11}H_{12}O_3$
Malvidin	$C_{17}H_{15}O_7$
alpha-Linolenic acid	$C_{18}H_{30}O_2$
Octadecanoic acid	$C_{18}H_{36}O_2$
5,7,4′-Trihydroxy-3,6-dimethoxyflavone	$C_{17}H_{14}O_7$
Anethole	$C_{10}H_{12}O$
Procyanidin C1	$C_{45}H_{38}O_{18}$
Eriocitrin	$C_{27}H_{32}O_{15}$
NarinGin	$C_{27}H_{32}O_{14}$

Methoxyphenylacetic acid	$C_9H_{10}O_3$
Patuletin 3-O-(2″-feruloylglucosyl)(1- > 6)-[apiosyl(1- > 2)]-glucoside	$C_{43}H_{48}O_{25}$
Kaempferol	$C_{15}H_{10}O_6$
Bisdemethoxycurcumin	$C_{19}H_{16}O_4$
Prodelphinidin dimer B3	$C_{30}H_{26}O_{14}$
Isorhamnetin 3-O-galactoside	$C_{22}H_{22}O_{12}$
Episesamin	$C_{20}H_{18}O_6$
Sinapaldehyde	$C_{11}H_{12}O_4$
Sinensetin	$C_{20}H_{20}O_7$
Lariciresinol-sesquilignan	$C_{20}H_{36}O_{10}$
Conidendrin	$C_{20}H_{20}O_6$
alpha-Ionone	$C_{13}H_{20}O$
3-Hydroxyphloretin 2′-O-glucoside	$C_{21}H_{24}O_{11}$
Apigenin 7-O-glucoside	$C_{21}H_{24}O_9$
Salicylaldehyde	$C_7H_6O_2$
Glycitin	$C_{22}H_{22}O_{10}$
Chrysoeriol 7-O-glucoside	$C_{22}H_{22}O_{11}$
Procyanidin B2	$C_{30}H_{26}O_{12}$

Ferulic acid 4-O-glucoside	$C_{16}H_{20}O_9$
Arctigenin	$C_{21}H_{24}O_6$
Arachidic acid	$C_{20}H_{40}O_2$
Hesperidin	$C_{28}H_{34}O_{15}$

Table S4. Most representative matched metabolites in *P.avium* extract (VCE) and their formulas. Compounds with an area % <0.1 were considered traces.
